# Legal Personhood for Animals: Has Science Made Its Case?

**DOI:** 10.3390/ani13142339

**Published:** 2023-07-18

**Authors:** Michelle C. Pardo

**Affiliations:** Duane Morris LLP, Washington, DC 20001, USA; mcpardo@duanemorris.com

**Keywords:** legal personhood, habeas corpus, nonhuman animals, sentience, American legal system, animal rights, zoological facility, mirror test, animal welfare

## Abstract

**Simple Summary:**

The commentary discusses the recent movement by animal rights organizations to secure legal personhood for animals in various courts in the United States. Laws, regulations, and legal jurisprudence are often influenced by scientific and societal developments. However, legal challenges on behalf of animals residing in zoological facilities using habeas corpus, a mechanism traditionally used by prisoners to challenge conditions of confinement, have been unsuccessful in each state and federal court that has considered the issue. Courts have been unwilling to include nonhuman animals within the meaning of “person” or afford them the legal rights and protections that humans receive in the American legal system. An analysis of these legal personhood decisions reveals that courts have rejected animals’ complex cognitive abilities as a justification for an extension of the law. Courts have been careful to state that societal-changing legal developments must originate in the legislatures, not the courts, and emphasize that significant societal and ethical issues may occur if animals are extended the legal rights and protections afforded to humans.

**Abstract:**

The use of Latin in identifying an organism’s genus and species is likely familiar to scientists and zoological professionals, but a traditional legal doctrine, known as habeas corpus (meaning “you have the body”) may not have obvious applicability to nonhumans in the animal kingdom. In recent years, animal rights organizations have utilized the habeas corpus doctrine as a basis to bring legal challenges on behalf of nonhuman animals to expand “legal personhood” to them. These lawsuits, which have focused on species such as nonhuman primates and elephants, seek to challenge the “confinement” of animals in zoological institutions and by private owners, much like a prisoner or other detainee. The small but vocal animal legal personhood movement bases its argument on the fact that elephants and nonhuman primates are highly sentient and have complex cognitive characteristics. Proponents of legal personhood for animals have argued that the common law has progressed and expanded over the years as societal norms and conditions have changed and, much like the law has expanded to afford women and persons of color legal rights and protections, so should the law expand to treat animals the same as humans. Despite these efforts, to date, no court in the United States has accepted this invitation. This article summarizes key legal challenges and decisions to date in the United States, examines how science and societal conditions have influenced the law, and analyzes the reasons why legal personhood for animals so far has been viewed as a “bridge too far” in the American legal system.

## 1. Introduction

In recent years, a small but vocal arm of the animal right’s movement has gone beyond advocating for the broadening of animal protection laws and their enforcement and influencing people to cease using animals in areas such as the food supply, zoological exhibits, and animal research. Instead, it has embarked on a litigation strategy to fundamentally change how animals are viewed by the American legal system. The quest for “legal personhood” for animals, in which animals are afforded the same legal rights as people, was born of the belief that animals’ status as “property” in the law has prevented humans from properly serving animals’ well-being. Advocates for animal legal personhood have utilized the centuries-old doctrine of habeas corpus and other “next friend” procedures as the basis to bring legal challenges on behalf of animals to secure their liberty and equality in society. 

A foundational premise of the argument for animal personhood focuses on science, sentience, and the complex, cognitive abilities that certain species possess. Developments in scientific research have demonstrated that certain animals, such as elephants and nonhuman primates, are sentient, cognitively complex beings and display qualities such as self-awareness and autonomy and other displays of emotions. These abilities are important cornerstones of the human experience. In addition to scientific developments, animal personhood proponents have argued that much like the common law has progressed and expanded over the years to, for example, afford women and persons of color legal rights and protections, so should the law expand to treat animals the same as humans. Despite these efforts, to date no court in the United States has accepted this invitation. 

Even crediting the myriad of scientific research advances in veterinary medicine, animal welfare, cognition, and behavior, how far can (or should) science influence the law? Does the future of animal personhood depend upon whether scientific research delivers more similarities between human and animal cognition and capabilities? Could a single scientific finding be the catalyst that shifts the animal personhood argument from the metaphysical to the mainstream? Additionally, is legal personhood for animals the solution for those who think that granting such rights is the only means—or even an effective means—to achieving optimal animal welfare? Can science answer the question of what environment is best for an animal that does not live in a wild habitat? And, even if legal personhood could ultimately advance an aspect of animal welfare, is society ready to upend the concept of what is a “person” and accept the intended and unintended consequences of such a seismic shift? 

This commentary summarizes the legal landscape of the animal personhood movement, including an overview of legal challenges and decisions. It also examines how science and societal conditions have influenced the law and whether any of these factors should upend how the American justice system views and treats animals and why, to date, animal personhood has so far been viewed as a “bridge too far” in the federal and state courts. 

## 2. Discussion

### 2.1. The Animal Personhood Movement

Many individuals active in the zoological community and animal industries have long witnessed animal rights and animal activism in action, sometimes being the direct recipient of activists’ efforts to prohibit or change their ownership rights, livelihoods, passions, and missions as they relate to animals. For decades, animal rights groups have focused on changing public opinion about how people use and interact with nonhuman animals (hereinafter “animals”). Much of these agendas have focused on public education and changing public opinion; legislation at the federal, state, and local levels; and targeted litigation. Enforcement of animal welfare laws, however, is often limited to state and federal prosecutors, as most animal welfare and protection laws do not have a comparable civil cause of action that individuals and organizations may use to address a perceived or actual animal welfare issue. In the last decade, however, animal activists have pursued “legal personhood” for animals or “animal personhood” cases in various state and federal courts. While they have taken various procedural forms, these cases aim to change the entire legal paradigm of how the law views animals—from “property”, capable of being owned, to “people”—by granting animals the same substantive and procedural rights as human beings. These cases have garnered much commentary and criticism, being labeled as “frivolous” [1] to “radical” [2] to creating “a deep philosophical muddle” [3]. Nonetheless, they have fueled considerable debate within the zoological and activist communities and in the court of public opinion. 

### 2.2. Elephants and the Mirror Test

Metaphysical poet and scholar John Donne called the elephant “nature’s great masterpiece” [4], an observation which is unlikely to cause debate. Elephants, part of the popular group of “charismatic megafauna,” are among the species that have a high cultural and societal value [5]. The popularity of a species can make them a particularly good ambassador for conservation efforts and other important projects, but they also can introduce bias into scientific studies and biodiversity protection [6]. That many wildlife conservation choices are based on subjective and not scientific grounds illustrates that human biases toward certain animals may directly impact how society values and cares for them [6] (p. 234). 

In 1970, psychologist Gordon Gallup had developed the mirror or mirror self-recognition (MSR) test as a method for determining whether animals are capable of self-recognition [7] and later was the first to hypothesize about the connection between MSR and empathy [8]. In 2006, researchers published a study on Asian elephants at the Bronx Zoo in New York City, reporting that four subject elephants recognized themselves in the mirror and one—an elephant named “Happy”—passed the so-called mirror or “mark” test [9]. The decades-old test involved placing a visual “mark” on the animal’s skin in a place that they could not see without the mirror’s assistance, then studying if the animal would notice and touch the mark [9]. The mirror test revealed that the elephant’s reactions to the mirror were self-directed responding behaviors, indicating that elephants are self-aware and can distinguish oneself from another, a behavior that has been “exceedingly rare in the animal kingdom” and shown in a limited number of species to date (humans, chimpanzees, and to some extent, dolphins) [9]. Self-recognition underlies social complexity and has been linked to human-like qualities, such as empathy and altruism [8]. For example, elephants have been observed assisting (lifting up) an injured conspecific [10] and displaying grief-like behavior [11]. While Happy’s passage of the mirror test demonstrates that elephants have the capacity for mirror self-recognition, it is not the case that every individual will pass the test, as was the case with the other Bronx Zoo elephants. 

But what exactly is the significance of Happy’s MSR results? Happy’s result demonstrated a single elephant’s capacity for cognitive capabilities that are shared with humans. As those in the zoological community are aware, captive animal welfare is dependent upon a caregiver’s ability to satisfy physical and mental needs. Basic husbandry needs, such as food, water, shelter, and medical care, must be paired with a stimulating environment, such as through environmental enrichment, which aims to introduce complexity that the species may have experienced in the wild [12]. It is commonly understood that species with higher degrees of cognitive abilities, such as chimpanzees and elephants, may require more complex stimulation and enrichment than other species in order to maintain good welfare. Happy’s mirror test results would, therefore, suggest that she requires a habitat and care that is complex enough to support her cognitive abilities and needs. In defining the needs of a captive species, however, how does one make the leap from the need for enhanced environmental enrichment to legal personhood? In 2008, the researchers likely did not realize that their research would serve as a basis to “open the personhood door” and posit a restructuring of our entire legal system [13] (p. 538).

### 2.3. Habeas Corpus Doctrine: Testing the Legality of Animals in Human Care

The Nonhuman Rights Project (“NhRP”) is a U.S.-based organization founded by an animal protection lawyer in response to what he saw as the limitations of animal welfare laws [14]. Calling itself “the only civil rights organization in the United States dedicated solely to securing rights for nonhuman animals”, NhRP sought to change the legal status of animals in the American legal system from “things” that are treated as property in the law to legal “persons” [14]. A significant legal stumbling block for NhRP is that there is no civil cause of action that provides a basis to sue an owner of an animal in order to gain that animal’s “freedom” from its owner or habitat. NhRP decided to use the centuries-old procedure of habeas corpus, also known as the “Great Writ”, as a means to have a U.S. court determine whether an animal is being illegally “imprisoned” and grant its release from such imprisonment. Translated from Latin, habeas corpus “you have the body”, [15] has been an important procedural mechanism to safeguard a person’s individual liberty. In a habeas proceeding, the defendant (often a prisoner) is brought before the court to determine whether the defendant’s custody is legal or not.

### 2.4. Chimpanzees in Court

NhRP’s quest for animals to have legal personhood started with its chimpanzee “clients” before progressing to elephants. In 2013, NhRP filed its first court cases in the New York state court system on behalf of four chimpanzees living in captivity in the state (with private owners and a research facility), including NhRP’s “first client”, a chimpanzee named “Tommy”, which is profiled here (“*Lavery I*”) [16]. The chimpanzees’ petitions demanded “recognition of the legal personhood and fundamental right to bodily liberty of autonomous nonhuman animals living in captivity” and the chimpanzees immediate release to a sanctuary [14]. Tommy’s petition was premised on the representation that “[c]himpanzees are autonomous, self-determined, self-aware, intelligent, and emotionally complex” and that “cognitively they resemble human beings” [17] (p. 1). If Tommy was found to be covered by the common law writ of habeas corpus, the person illegally confining the “prisoner” would then bear the burden of proving the imprisonment of Tommy was legally sufficient. 

Much of NhRP’s petition cites to physiological, developmental, and behavior similarities that chimpanzees share with human beings [14]. While recognizing that chimpanzees are not human beings, the petition argued that these intellectually complex qualities were sufficient to grant chimpanzees “personhood” and the rights afforded to person in the law [17] (p. 2). NhRP further reasoned that its ability to create a trust for Tommy under Section 7-8.1 of New York’s Estates, Powers, and Trusts Law, which identified the chimpanzee as a beneficiary and “legal person”, was evidence that legal personhood could be extended to include nonhuman animals [17,18] (pp. 49–52). It is this foundational argument—that a “legal person” is not a biological concept (and therefore not limited to those who are human beings) but merely a “term of art”—that once excluded “human fetuses, human slaves, Native Americans, women, corporations, and other entities” that urged an evolution of the law from animals as “thing” to “person” [17] (pp. 40; 42). As NhRP argued “[w]hen justice requires, New York courts refashion the common law with the directness Lord Mansfield displayed in *Somerset v. Stewart*, which held slavery ‘so odious that nothing can be suffered to support it but positive law’” [17] (p. 64 (quoting 98 Eng. Rep. 499, 510 (1772)). 

Another pillar of NhRP’s personhood argument is that human infants and adults with physical and intellectual disabilities may not be able to uphold legal duties and responsibilities, but they are nonetheless afforded legal rights of “persons” [17] (pp. 48–49). Law Professor Richard L. Cupp, Jr., who has written extensively on animals and legal personhood as well as submitted amicus curiae (friend of the court) briefs opposing animal personhood, on which courts have relied, has analyzed the connection between rights and responsibilities of children and cognitively impaired humans, referred to by philosophers as “the argument from marginal cases” [19,20]. That argument is based on the concept that if less capable (so-called “marginal”) persons (such as children or those with cognitive impairments) are assigned legal rights, other intelligent animals with greater abilities or autonomy than “marginal” humans should be afforded the same legal rights as these persons [20]. Cupp’s survey of the law and his parallel argument to the courts found that the rights of children are “anchored in their belonging to the human community where moral agency, sufficient to be held accountable in society’s legal system, is the norm” [20] (p. 45–48). The notion of “belonging to the human community” has been criticized as being a form of “speciesism” or assumption of human superiority. As Cupp noted, legal scholar Richard Posner recognized the “superior claim of the human infant than of the dog” is part of a “moral intuition deeper than any reason that could be given for it and impervious to any reason that anyone could give against it.” [20] (p. 29–30). Posner’s statement may seem unapologetic and a rather perfunctory way of resolving the argument, but the notion of membership in the human species as being categorically different and thereby requiring different treatment in society’s laws is a concept that U.S. courts have embraced as a basis to reject the extension of the law sought by animal personhood cases. 

Tommy’s petition (as well as the other chimpanzees) was rejected by the trial court, which found that habeas corpus did not apply to chimpanzees [21]. The appellate court affirmed that decision, noting that “[n]eedless to say, unlike human beings, chimpanzees cannot bear any legal duties, submit to societal responsibilities or be held legally accountable for their actions” [21] (p. 152). The court also soundly rejected the argument that human beings lacking capacity to exercise their own rights provides a gateway to animal personhood, stating: “[t]hese differences do not alter our analysis, as it is undeniable that, collectively, human beings possess the unique ability to bear legal responsibility” [21] (p. 152, n.3). In short, animals and people were sufficiently distinct in the eyes of the legal system such that they had materially different status or “rights”.

A procedurally tedious history of litigation followed with another round of habeas corpus petition and appeals brought on behalf of Tommy (“*Lavery II*”), which ultimately concluded with the New York appellate court denying that chimpanzees are entitled to legal personhood and bodily liberty rights [22]. While joining in the decision to deny NhRP’s request to appeal to the highest court of review in New York, Judge Eugene Fahey authored his own concurring opinion, in which he called out the “inadequacy of the law as a vehicle to address some of our most difficult ethical dilemmas on display in this matter” and cited the issue of whether a nonhuman animal has person-like rights “profound and far-reaching” [22] (p. 1059) (Fahey, J., concurring in part). Stated simply, Judge Fahey observed: “‘[w]hile it may be arguable that a chimpanzee is not a ‘person,’ there is no doubt that it is not merely a thing“ [22] (p. 1059). 

### 2.5. The Case for Elephants and “Happy” 

Notwithstanding Judge Fahey’s minority opinion, NhRP fared no better in its quest for legal personhood in Connecticut Superior Court after a two-year legal challenge aimed at liberating three Asian elephants (Beulah, Minnie, and Karen) from the Commerford Zoo [23]. The first habeas corpus petition filed on behalf of elephants, however, received a much harsher rebuke than delivered by the New York courts, with the Connecticut court calling the petition “wholly frivolous on its face in legal terms” [24]. As a threshold matter, the Connecticut court determined that NhRP had failed to allege that it possessed any relationship with the elephants, such that it could serve in court as “next friend” on the elephants’ behalf and therefore had no standing to sue [25] (p. *3). The court went further, finding that because NhRP’s theory was not based on any legal authority that granted elephants the same rights as humans, it was unwilling to “forge new law” [25] (p. *5). All subsequent requests for relief and to reverse the trial court’s decision, including a motion for en banc (by the full court) reconsideration, were all denied [25].

Perhaps the media’s fascination with Happy’s landmark results on the mirror test are why her habeas matter, brought by NhRP (on Happy’s behalf) against the zoological society that operates the Bronx Zoo in New York and its director, garnered much more widespread attention [26,27,28]. Happy’s habeas petition began where Tommy’s left off—invoking Court of Appeals Judge Fahey’s description of the animal personhood issue as “a deep dilemma of ethics and policy that demand our attention” and arguing that a refusal to grant Happy’s petition would create “manifest injustice” [29] (p. 3). However, where the chimpanzee lawsuits involved animals in private ownership and in a research facility, Happy had been living at a USDA-licensed and accredited zoo. NhRP also cited to intervening, foreign legal decisions that had been decided since the chimpanzee matters as legal authority to support its petition for relief. In the first, an Argentine court issued a writ on behalf of an orangutan named Sandra [30]. In the second, another Argentine court recognized that a chimpanzee named “Cecilia” is a “non-human person” and ordered her released from a zoo to a sanctuary in Brazil [31]. Rejecting the claim that Cecilia could not avail herself of the habeas corpus doctrine because she was not human, the Argentine court recognized that “societies evolve in their moral conducts, thoughts and values” and concluded that legally classifying “animals as things is not a correct standard” [32] (pp. 19–20; 23–24). Like Tommy, Happy was the beneficiary of a trust under New York law, created by NhRP itself to provide for her care if the requested relief—transfer to an elephant sanctuary—was granted [30] (p. 14). However, despite nascent legal decisions in other countries, the United States has yet to follow the Argentine or any other foreign tribunals. 

NhRP’s brief in support of its petition includes a litany of capabilities possessed by elephants—from planning and communicating to advanced working memory skills to cognitive mapping skills to emotions and lasting memories [32]. However, perhaps the most referenced capability in NhRP’s quest for Happy’s personhood was her ability to exhibit mirror self-recognition [30] (p. 39). NhRP reasoned that MSR is a “key identifier” of self-awareness, and therefore, Happy’s MSR must mean that she is capable of a mental representation of herself as a separate entity from others. According to NhRP, this concept of “self” and “awareness of death”, which NhRP notes elephant researchers have observed in the wild, underpin aspects of the human experience, thereby justifying the extension of human rights to Happy [30] (pp. 39–40). 

While the Bronx County Supreme Court (New York’s trial level court of general jurisdiction) took at face value that Happy and potentially other elephants shared “numerous complex cognitive abilities with humans” and passed the MSR, the court, citing the failed *Lavery* habeas petitions, denied Happy’s petition, finding that animals are not “persons” entitled to the rights and protections of the writ [33]. The Appellate Division affirmed that decision [34]. NhRP filed a petition for discretionary review with the Court of Appeals in New York, the highest court in the state. What followed next was an extraordinary amount of briefing, both by NhRP, the Bronx Zoo, and a myriad of amicus briefs from NhRP’s purported elephant experts, to legal academics, to members of the veterinary and zoological communities, to pro agriculture and biomedical research industry groups and organizations. Happy’s case drew supporters and opposition from all corners of society, including those who argued that Happy’s MSR and other capabilities required a creation of new legal precedent [35]. 

After extensive briefing and a virtually-broadcasted oral argument, the Court of Appeals of New York held that an elephant was not a person with liberty rights and affirmed the denial of Happy’s habeas corpus petition [36] (pp. 565–566). “Unlike the human species, which has the capacity to accept social responsibilities and legal duties, nonhuman animals cannot—neither individually nor collectively—be held legally accountable or required to fulfill obligations imposed by law” [36] (p. 572). The court rejected the argument that extending habeas relief to nonhuman animals was the “natural progression” from its extension to “abused women and children and enslaved persons” [36] (p. 571). Notably, the court pointed out that granting the petition would not guarantee that Happy—who had been living at the zoo for 45 years—is free from captivity: “[t]he fact that the greatest relief which could be afforded Happy is a transfer between lawful confinements demonstrates the incompatibility of habeas relief in the nonhuman context” [36] (pp. 566; 572). Importantly, the court recognized that “no one disputes that elephants are intelligent beings deserving of proper care and compassion”; these capabilities and needs, however, did not transform their status in the law from “property” to “person” [36] (p. 566). 

Importantly, the court’s majority decision also focused on the “elephant in the room”; if it were to grant animals the same rights as people, what would the impact be on society [36] (pp. 573–574)? In the court’s view, this would have “an enormous destabilizing effect on modern society”, including to agriculture, property rights, medical research, and even pet ownership [36] (p. 573). It further stated that such a decision would create the ultimate slippery slope by “requir[ing] us to upend the state’s legal system to allow highly intelligent, if not all, nonhuman animals the right to bring suit in a court of law” and would force “owners of numerous nonhuman animal species—farmers, pet owners, military and police forces, researchers, and zoos, to name just a few” to defend themselves in those actions [36] (p. 574). 

However, two of the judges rejected the majority’s reasoning and its practical considerations and authored lengthy dissenting opinions, [36] (pp. 577–642) (Wilson, J. and Rivera, J. dissenting), in part arguing that a “functional intelligence” test could limit personhood rights to those more intelligent species for which confinement is more problematic [36] (pp. 574–575). In response, the majority stated: “the dissenters’ wholly unsatisfactory attempt to distinguish ‘domestic’ animals from elephants. simply because they live comfortably among humans or are supposedly genetically predisposed to confinement is divorced from practical reality, devoid of support, and demonstrates the contradictory foundation on which their analyses are built” [36] (p. 575). In addition, as to science’s role in sorting out what species are deserving of personhood rights, the court predicted that “giving a court authority to interpret the relevant ‘science’ would have perilous implications far beyond the issues here” [36] (p. 575). In short, the highest court in New York delivered a resounding rebuke to the animal personhood issue. Unsurprisingly, NhRP lauded the dissenting opinions and indicated that they looked forward “to citing the dissents in our elephants’ rights cases underway in California and in the new cases we’ll file across the US and in other countries in the coming months” [37]. 

### 2.6. Animal as Plaintiff—“Next Friend” Cases

While the habeas corpus doctrine has been the favored procedural vehicle for NhRP and its animal “clients” to get into court, other animal activists have pushed the legal envelope by arguing that animals have the right to participate as plaintiffs in court and are covered by federal laws protecting a person’s right to intellectual property. In 2015, animal rights group People for the Ethical Treatment of Animals (PETA) brought a “next friend” case in California federal court on behalf of a crested macaque [38]. In 2018, the Animal Legal Defense Fund (ALDF) represented an American Quarter Horse (re-named “Justice” for the litigation) and Justice’s human “next friend” in an Oregon state case [39]. Both cases introduced “animal as plaintiff” in a slightly different procedural manner, but neither animal rights group was successful and the lawsuits garnered significant criticism [40,41]. The PETA lawsuit, discussed below, resulted in an uncharacteristically critical Ninth Circuit decision.

PETA’s federal lawsuit argued that a macaque had a Copyright Act claim against a wildlife photographer [42]. While photographing wildlife in a reserve on the island of Sulawesi, Indonesia, photographer David Slater’s camera was swiped by a curious macaque (“Naruto”), who snapped “selfies” [42] (p. 420), including a wide-grinning one that subsequently garnered headlines. Ironically, in the published book, the author described Naruto as “[p]osing to take its own photograph, unworried by its own reflection, smiling. Surely a sign of self awareness” [42] (p. 420). When Slater published the “monkey selfie” in his book, in which he identified himself and the publisher as the copyright owners of the monkey selfies, PETA, acting as “next friend” of the monkey, brought a federal lawsuit in the Northern District of California on the monkey’s behalf to enforce the Copyright Act [42]. Finding that the monkey had no standing to sue for a violation of the Copyright Act, the district court dismissed the case [41] (pp. *3–4). PETA appealed to the Ninth Circuit, but after an oral argument and before a decision had issued, PETA settled the case, although Naruto evidently was not a party to the settlement [42] (p. 421, n.3). In an unusual turn, despite the settlement, the Ninth Circuit decided to issue a decision, which was highly critical of PETA and its motives in bringing the lawsuit. 

The Ninth Circuit panel criticized PETA for claiming “next friend” status to the monkey, as PETA had failed to allege any facts to support the requisite significant relationship between a “next friend” and a “real party in interest” [42] (pp. 421–422). While the court recognized the appropriateness of “next friend” lawsuits on behalf of habeas petitioners and minors or incompetent persons, it declined to extend this status to animals, absent express authorization from Congress [42] (p. 422). The Ninth Circuit reluctantly found that Naruto had Article III standing (the ability to sue generally) based upon *Cetacean Community. v. Bush*, an earlier, binding decision brought on behalf of “all of the world’s whales, porpoises and dolphins”, a case that had alleged physical injuries from the Navy’s sonar systems [43]; [42] (p. 425, n.7). The *Naruto* court criticized that decision as “incorrectly decided” [42] (p. 425, n.7). While technically bound by the *Cetacean Community* decision, the *Naruto* court urged the Ninth Circuit to reexamine that case [42] (pp. 423; 425, n.7). As to statutory standing under the Copyright Act, the court found that Congress has never provided that any animal may sue in their own name in federal court, and there is no aspect of federal law that has recognized this right [42] (p. 425, n.7). 

The decision is notable, not just because of its unique facts, but because the Ninth Circuit delivered a harsh rebuke to PETA—first for settling and “abandoning” Naruto and his case, presumably “to prevent the publication of a decision adverse to PETA’s institutional interests” [42] (p. 421, n.3). While citing PETA’s mission, the court wrote: “[p]uzzlingly, while representing to the world that ‘animals are not ours to eat, wear, experiment on, use for entertainment, or abuse in any other way”, PETA seems to employ Naruto as an unwitting pawn in it ideological goals” [42] (p. 421, n.3). The court found this “institutional interest” fell short of the “significant relationship” that a next friend must have to represent a litigant [42] (p. 421, n.3). As in the state habeas cases referenced supra, the fact that corporations and unincorporated associations are afforded legal rights to sue did not persuade the court to extend such rights to animals. Drawing a parallel to former U.S. Supreme Court Chief Justice William Rehnquist’s findings in *Lenhard v. Wolff* [44], the court quoted: “however worthy and high minded the motives of ‘next friends’ may be, they inevitably run the risk of making the actual defendant a pawn to be manipulated on a chessboard larger than his own case” [42] (p. 422)

The 20-page concurrence was even harder on PETA, raising public policy grounds for not allowing next friend standing for animals. Calling PETA’s case “frivolous”, the concurring opinion also observed that PETA’s real motivation in this case was to “advance its own interests, not Naruto’s” [42] (p. 437, n.11) (Smith, J., concurring). The concurrence also noted that: “[p]articipation in society brings rights and corresponding duties. Are animals capable of shouldering the burden of paying taxes? Should animals [be] liable for intentional torts as well?” [42] (p. 432, n.6) (Smith, J., concurring). Unfortunately for PETA, the case ended with the court ordering PETA to pay Slater and the wildlife publication’s appellate attorney’s fees [42] (p. 427). Whether to advance ideological goals or simply to garner publicity for fundraising, the *Naruto* case is a cautionary tale about how a group may “use” an animal in litigation—which the Ninth Circuit strongly rebuked. 

### 2.7. Legal Personhood for Animals—Does Science Know Better?

For all of their zeal, animal personhood advocates to date have not been able to convince a U.S. court that scientific advancements regarding animal cognition justify a reinvention of the American legal system. This is despite the fact that society may view animals differently than it did decades ago. This is not the first instance in which a perceived “gap” between science and the law exists. While human and veterinary medicine may be quick to adapt scientific research and advancements into clinical practice, statutes, regulations, and judicial interpretations of the same evolve develop at a much slower pace. One may say that scientific advancements and legal jurisprudence are based on incompatible philosophies. Legal decisions are based on the principle of stare decisis—“to stand by things decided” [45], meaning that courts will adhere to previously decided precedent in making decisions. Indeed, many legal decisions are based upon interpretation of the United States Constitution—a document written in 1787—and the “framer’s” intent. 

On the other hand, science and technology are ever advancing, evolving and built on objective, reproducible research and data. To cite just a few examples, federal regulations, such as those implementing safe levels of chemicals in our food, water, and air supplies, are constantly being updated to accommodate new data and scientific advancements. Anti-abortion law advocates have relied upon advancements in science to bolster their argument of when a fetus can feel pain or gain certain functions as a basis to change laws [46]. Advancements in DNA science have revolutionized how physical evidence is analyzed and utilized in criminal prosecutions [47]. Advancement of scientific understanding about animal cognition, capabilities, and behavior has certainly advanced animal welfare laws. However, does the future of animal personhood depend upon whether scientific research delivers more similarities between human and animal cognition and capabilities? Could a single scientific finding be the catalyst that shifts the animal personhood argument from the metaphysical to the mainstream? In addition, in the case of such a scientific finding, can we be sure that they are being presented to the courts free from politicization, philosophical agenda, and misinformation that unfortunately has plagued the presentation and discussion of science in the public domain in recent years [48]? The science of animal welfare is unlikely to be immune from such politicization. 

### 2.8. Re-Thinking the Mirror Test

The New York Court of Appeals did not accept that science and data regarding elephant cognition justified providing animals with the legal rights of humans and warned that courts should not be the arbiter’s of science and species [36] (p. 575). But what if the mirror test had convinced the court that Happy had a right to be in court and seek her liberation from her long-standing caregivers and zoological home? One would hope such a monumental decision would be based on, at a minimum, an irrefutable scientific principle. In addition, what if this scientific benchmark for assessing self-awareness—a foundational requirement for species that are considered for legal personhood—is fundamentally flawed? Could a seismic change to our legal system be prompted by the ubiquitous scientific test delivering incongruous results? 

In 2012, evolutionary biologist Alex Jordan began studying how cleaner wrasse (*Labroides dimidiatus*) fish reacted to a mirror placed in their tanks [49]. When the “mark” was administered (by superficially injecting colored dye into the fishes’ throats), the fish appeared to notice the mark in the mirror and then scratch it in the sand. However, the fish made no such responses in the absence of the mirror [49,50]. This led Jordan and his fellow researchers to believe that either the scientific community would have to accept that the small-brained fish were self-aware or rethink the meaning of the mirror test entirely [49]. In their published report, Jordan and his co-researchers posited the following: what do the cleaner fish results mean for our understanding of animal intelligence and the mark test as a metric for animal cognitive abilities [49]? 

The research was quite polarizing, and garnered both positive and negative reviews including a “primer” authored by the report’s Academic Editor (de Waal), which presented a “complementary expert perspective” on how this case study should be interpreted in the context of evidence for and against self-awareness in other animals [51]. The main point of criticism was that the fishes’ self-scraping behavior may not be the same as self-exploration with hands or a trunk and therefore not sufficiently indicative of self-awareness [51]. De Waal noted that the mirror mark test “has encouraged a binary view of self-awareness” and compared the test to a “Big Bang theory”, noting that science needs “a much larger test battery, including nonvisual tasks, to develop a full understanding of how other species position the self in the world [51]. 

Jordan’s study (and its follow-up that enhanced the study methodology and results and addressed some of the criticism [50]) had been published before New York Court of Appeal’s decision regarding Happy, but it did not find its way into the legal briefings. However, de Waal’s comments raise a relevant inquiry: when courts are considering an animal, such as an elephant or chimpanzee’s “rights” in society, how is one supposed to discern this from “binary” or isolated pieces of scientific data and observation? In other words, who or what decides what legal status makes Happy, well, happy? 

### 2.9. The Anthropomorphism Factor

When considering the argument that animals are entitled to body autonomy and legal rights, one also cannot discount the role that anthropomorphism plays in NhRP’s or any animal rights litigant’s perspective. Science has traditionally cautioned against explaining animal behavior with human terms and descriptions. Anthropomorphism is defined as the “tendency to attribute human forms, behaviors and emotions to non-human animals or objects” and often to project human needs onto them [52]. This practice may lead to misinterpretations of an animal’s needs or behavior, with attendant negative effects [52]. However, where anthropomorphism may be a constructive tool in studying animal behavior, it has been criticized as not being based on a well-developed scientific system and little more than “informal folk psychology” [53] and, therefore, a faulty basis for groundbreaking legal decisions. 

Sitting on the opposite side of the spectrum is “anthropodenial”—meaning the rejection of shared characteristics between human and animals, as coined by primatologist Frans de Waal [54]. To deny any shared behavior or awareness between humans and animals—a “willful blindness” to any human-like characteristics of animals—is surely not the answer and may be why de Waal argued that a judicious form of anthropomorphism is not a problem. That the line between “proper” anthropodenial and anthropomorphism has likely shifted over decades of scientific research and discovery does not make the puzzle easier to solve. 

A key issue with the animal personhood movement is the risk that those exercising the legal rights—ostensibly on behalf of animals—are really acting based on a human’s wants and needs rather than what is in the animal’s best interest. Viewing an elephant and describing her as “lonely” or “sad” may be used to describe an animal who, due to circumstance, such as death of a conspecific, is living alone. However, this observation, in and of itself, is not based on a well-developed scientific system and, indeed, may be highly influenced by the observer’s training, background, or personal view of animals in captivity. NhRP describes Tommy and Happy as its “clients” [14], but if Happy were permitted to choose her advocate, would she prefer her dedicated caregiver and decades-long familiar environment at the Bronx Zoo? Would she want a court to order her to move out of her familiar environment and away from her long-term caregivers? Or, would Happy prefer her destiny be directed by an animal rights activist who is taking up her legal case as a means toward the much larger goal of fundamentally changing what a society may or may not do with an animal? A legal case and ultimate disposition of the animal is driven by a human, as it must be because animals lack the capacity to manage their affairs in a lawsuit. Because of this inevitable role, human biases and philosophical goals risk taking priority over the best interests of the individual animal. 

The concurring opinion in the *Naruto* decision captures the dilemma of letting humans who are opposed to animals living in zoological or other human care take the lead in enforcing animals’ “rights”. How do we as humans know what another species truly desires?

Do animals want to own property, such as copyrights? Are animals willing to assume the duties associated with the rights PETA seems to be advancing on their behalf? Animal next-friend standing is materially different from a competent person representing an incompetent person. We have millennia of experience understanding the interests and desires of humankind. This is not necessary true for animals. Because the “real party in interest” can actually *never credibly articulate its interests or goals*, next-friend standing for animals is left at the mercy of the institutional actor to advance its own interests, which it *imputes* to the animal or object with *no accountability*”.

[42] (p. 432) (Smith, J. concurring in part)

As the courts and commentators have noted, there may be little debate from a next friend or guardian ad litem as to what a child involved in a potential deprivation of rights or care situation actually needs. These adults share the human experience with the child and generally understand what is in their best interest. Notably, in child welfare and custody cases, courts still take great pains to preserve parent and familial ties and relationships, even if another environment may be objectively preferable. However, for an animal living in zoological care who has bonded with caregivers, experienced good health, reproduced successfully, and displayed species-specific behaviors, who is NhRP or any other activist group to step in and argue that the animal must be relocated out of her familiar environment and routines to a so-called better life? Such arguments are routinely made by animal personhood advocates, even where federal Animal Welfare Act regulations are all being complied with and the zoo subscribes to additional accreditation and certification standards by independent, third party animal welfare organizations. In that scenario, how would a court determine the best interests of the animal? With veterinary, behavior metrics, or other scientific tests? Or a more ideological rationale that the animal will better exercise her “autonomy” in a different, human-managed environment? 

Researchers have cautioned that imprecision and missteps in animal behavioral assessments can result in misdiagnosing the welfare state of an animal and lead to negative consequences [55]. For example, holistic definitions of welfare may be based on a multitude of factors but not describe the conditions under which one factor should “trump” another [55] (p. 76). Interpretive biases can lead to misconceptions about an animal’s needs. An animal receiving an incorrect diagnosis of a welfare issue may be subject to an intervention that is “unwarranted, harmful, or ineffective” [55] (pp. 80–81). For example, “if an animal were incorrectly identified as having poor welfare when it did not and a major change was prescribed as a solution, such as a significant enclosure modification or a dramatic change to the social group, there could be added discomfort during construction or potential incompatibility with social partners that could lead to a real decline in the individual’s welfare. These sorts of modifications may result from a misguided focus on ‘natural living’ opportunities for animals” [55] (p. 80). 

For as much as neuroscience has focused on the concept of neural plasticity and how fundamental it is for a species or an individual’s adaptation to change, this process varies widely from one mammalian species to another [56]. For an animal like Happy, who has lived for decades in the same place, could a well-meaning but ill-conceived belief that she should be moved to another facility create physical and emotional stress that she is incapable of adapting to? As the *Breheny* majority eluded to, getting this science wrong can indeed be “perilous” to the species. From the perspective of the animal, therefore, the “choice” of a “next friend” determining its legal fate is a high-stakes decision. Of course, neither Happy nor any other animal would actually get to choose its courtroom advocate. 

### 2.10. The Law’s Capacity to Influence Social Norms and Behaviors

Considering that the stated premise of the animal personhood movement was to improve and advance greater respect for animals and improved welfare, it is important to analyze whether a change in the legal status of animals could bring forth a marked improvement in animal welfare. For those who cannot conceive of animals exercising legal rights in the courtroom, could a change in the law nonetheless influence how people view and treat animals? Studies have shown that laws and social norms are interconnected and that laws may even change individuals’ perceptions of the prevailing social norm [57]. However, a law is only likely to affect social norms if it is viewed as “legitimate, fair and close to pre-existing social norms” [57]. Similarly, laws that come about by way of a referendum by the voters rather than by a central authority are often more effective in changing social norms and behavior [57], no doubt because they reflect a majority viewpoint of society. An illustrative example is the heated societal and legal debates surrounding gender, sex, and pronouns [58]. The gender/sex binary—that a biological female is a “woman” and a biological male is a “man”—is deeply-embedded in our society and many of our lived experience and psyches. Policies and laws that have tried to “de-gender” or “multi-gender” have been met with controversy and extreme resistance, thus aligning with the principle that laws which stray far from accepted social norms may not actually change human behavior [58]. 

Legal personhood for animals undoubtedly would be a radical change in the law that would not fit the “close to pre-existing social norms” category. Therefore, a legal decision that extends human rights to animals and allows animals to sue in court, or similar legislative action by the United States Congress or a state legislature, would seem to face an uphill battle in socializing people to the concept. It would be hard to conceive of a more radical shift in the law as one’s pet or cattle being able to sue them for their “confinement” in a dog kennel or pasture, or an animal rights group that wants to shut down your animal business because, in their view, the animals need “freedom”. Aside from changing the quality or type of habitat, the question remains: would animal personhood materially improve an animal’s life? Or, would many of these cases promote change based on ideology and not the best interest of the individual animal? Would it fundamentally change humans’ behaviors toward them? Because humans would still be responsible for animals’ daily care and housing, would a world with legal personhood look a lot like the status quo—or worse, subject animals to unnecessary and arbitrary changes in their habitats, caretakers, veterinary care, and familiar routines? 

Whether it is the court or the legislatures that decides to bestow legal personhood on an animal, the fact remains that the animal will need its (his? her? their?) “next friend” or a representative to exercise any of those rights on the animal’s behalf. Deciding who may be the best advocate for an animal may in and of itself become a legal battleground. Who, then, is the best choice of an advocate for any particular animal? Making that decision would necessarily require a court to wade into the quagmire of comparing competing philosophies and beliefs to decide what advocate will really act the best interests of the animal.

## 3. Conclusions

While accepting the fact that certain species have complex cognitive abilities and even share some capabilities similar to humans, U.S. courts have uniformly rejected the invitation to upend how state and federal legal systems view animals and have declined to grant nonhuman animals “personhood”. These decisions should not be viewed as turning a blind eye to animal welfare and the need to constantly improve how we treat and care for animals, but rather are practical in their approach. Significant societal and ethical issues may occur if animals are granted the same legal rights and protections afforded to humans. In particular, the courts could become unwilling referees in a philosophical debate between those who proudly and responsibly own, use, and display animals in their daily lives and those who oppose those views, and have instead insisted a material change in the law must come from the legislatures.

As much as science has contributed to animal cognition and improved welfare, animal personhood runs the great risk of misinterpreting what is in the individual animal’s best interest and elevating the philosophical and ideological needs of the human “next friend” above that of the animal that cannot speak in its own defense. The legal status quo may assign “property” rights to animals, but that does not relieve human caretakers from constantly improving the care it provides to nonhuman species. In the long run, the resources spent on fighting for animal personhood in the courts and the legislatures may be better directed toward enhancing the care of the species that are entrusted to our collective human responsibility or for important work involving their conservation.
